# PSYCHOPATOLOGICAL PROFILE OF SERBIAN RACE DRIVERS WITH PTSD

**DOI:** 10.1192/j.eurpsy.2023.2077

**Published:** 2023-07-19

**Authors:** C. G. Alexopoulos, J. M. Jovanovic Mirkovic, Z. Z. Jurinjak, B. M. Veljkovic

**Affiliations:** 1 Section of Cuprija, Anatomy and physiology; 2 Section of Cuprija, Anatomy, Physiology and Biochemistry; 3 Section of Cuprija, Social and Humanities, The Academy of Applied Preschool Teaching and Health Studies, Cuprija, Krusevac, Serbia

## Abstract

**Introduction:**

Race with cars is currently one of the most popular sports.

**Objectives:**

The aims of this study are establishing the profile of persons with posttraumatic stress disorder by using psychopathological dimensions – clinical scales (MMPI). Psychiatric measures (HAMD, HAMA, API) exploited to detect differences between acute and delayed type of PTSD on the level of depression, anxiety, and readiness for panic.

**Methods:**

The research included 30 drivers: 20 have reacted with acute and 10 with delayed onset of PTSD. Diagnosis criteria were DSM-V.

**Results:**

The scores on subscales at MMPI personality profile for acute and delayed type of PTSD, are much higher D (T=80.15, t=3.10, p<0.05) and Hy (T=79.25, t=3.02, p<0.05), in relate to normal (T=70). There was high level of appearing the structural correlates D (t=4.22, p<0.01) and HS (t=3.43, p<0.01) in delayed PTSD in relate to acute.

**Conclusions:**

There is a higher level of depression (HAMD: t=4.03, p<0.01) and of anxiety (HAMA: t=3.05, p<0.05). There is no statistical difference between acute and delayed PTSD, considering the panic. Whether running risk remains controversial.

Key words: PTSD, cars drivers, and psychological profile

**Disclosure of Interest:**

None Declared

